# Educational Needs and Preferences for Patient-Centered Outcomes Research in the Cystic Fibrosis Community: Mixed Methods Study

**DOI:** 10.2196/24302

**Published:** 2021-03-04

**Authors:** Emily M Godfrey, Traci M Kazmerski, Georgia Brown, Erin K Thayer, Laura Mentch, Molly Pam, Morhaf Al Achkar

**Affiliations:** 1 Department of Family Medicine University of Washington School of Medicine Seattle, WA United States; 2 Department of Pediatrics School of Medicine University of Pittsburgh Pittsburgh, PA United States; 3 Cystic Fibrosis Reproductive and Sexual Health Collaborative Seattle, WA United States

**Keywords:** cystic fibrosis, needs assessment, patient-centered outcomes research, training, education, team building, patient engagement

## Abstract

**Background:**

Cystic fibrosis (CF) is a rare, life-shortening, multiorgan disease, the treatment of which has seen significant increases in the life expectancy of those with CF. Many advances in CF care are thanks to the dedicated and active participation of people with CF as research participants. Unfortunately, most CF research teams still do not fully partner with people with CF or their caregivers.

**Objective:**

The aim of this study was to determine the interest, knowledge gaps, and desired format for patient-centered outcomes research (PCOR) training in the CF community.

**Methods:**

We surveyed patients, caregivers, researchers, research staff, and diverse health care providers via list servers and social media outreach about their knowledge of, experience with, and preferences for PCOR training components. We followed the survey with 3 small-group discussion sessions with 22 participants who completed the survey to establish consensus and prioritize key learning components of a PCOR training program. We summarized results using descriptive statistics.

**Results:**

A total of 170 participants completed the survey (patients/caregivers: 96/170, 56.5%; researchers/health care providers: 74/170, 43.5%). Among providers, 26% (19/74) were physicians/advanced practice providers, 20% (15/74) were nurses, and 54% (40/74) were from other disciplines. Among all participants, 86.5% (147/170) expressed interest in learning about PCOR, although training topics and training format differed between the patient/caregiver and researcher/health care provider groups. Before participating in PCOR, patients/caregivers wanted to understand more about expectations of them as partners on PCOR research teams (82/96, 85%). Meanwhile, researchers/health care providers desired information on how to include outcomes important to patients/caregivers (55/74, 74%) and the quality and impact of PCOR research (52/74, 70% and 51/74, 69%, respectively). Patients/caregivers were most interested in learning about the time commitment as a PCOR team member (75/96, 78%). Researchers/health care providers wanted to receive training about how to establish trust (47/74, 64%) and maintain confidentiality (47/74, 64%) when including patient or caregiver partners on the PCOR team. During follow-up discussions, participants emphasized the importance of addressing the traditional patient/caregiver and researchers/health care provider hierarchy by teaching about transparency, appreciation, creating a common language between the groups, and providing specific training on “how” to do PCOR.

**Conclusions:**

Our findings suggest CF community members are interested in PCOR. A high-quality training program would fill a current deficit in methodological research. This assessment identified the topics and formats desired and can be used to develop targeted training to enhance meaningful PCOR in CF.

## Introduction

Cystic fibrosis (CF) is a rare, life-shortening, multi-organ disease that affects approximately 30,000 patients in the United States [1]. CF impedes cell chloride protein channels leading to a cycle of impaired mucociliary clearance, inflammation, and infection in the respiratory tract, with related effects on the digestive, endocrine, immune, and reproductive systems. It can lead to severe respiratory and digestive problems as well as other complications such as infections and diabetes. Although CF traditionally affected children, today more than 50% of individuals with CF are adults with a median survival of almost 45 years [2].

People with CF have a long history of actively being involved as participants in research and thus have played a critical role in the medical advances seen in the CF community. The Cystic Fibrosis Foundation (CFF) is a large stakeholder organization that has led the effort to fund clinical research studies that advance the care and treatment of CF patients in the United States [3]. The latest breakthrough has been the development and recent Food and Drug Administration approval of a highly effective modulator therapy medication that corrects defective protein channels for 90% of those with CF 12 years and older, which is approximately 27,000 people in the United States [4].

Despite these achievements, patient participation in CF-related research has been mostly limited to involvement as participants enrolled in clinical trials. In the past two decades, the CF community and CFF have worked to deepen partnerships with patients through skill-building opportunities in quality improvement methods and the formation of patient and family advisory boards [5,6]. Another CF stakeholder organization, Cystic Fibrosis Research, Inc (CFRI), hosts an annual conference for researchers, clinicians, caregivers, and patients with CF and provides monetary support for CF research driven by stakeholders in the CF community [7]. Yet, despite these opportunities, partnership with people with CF and their families as equal players in research design and performance remains limited.

The Patient-Centered Outcomes Research Institute (PCORI), established in 2010 in the United States, now requires research engagement in all of its funded studies [8,9]. Patient-centered outcomes research fosters coproduction by engaging patients, caregivers, and other stakeholders as equal members on research teams [10]. Essentially, PCOR shifts power that typically rests with researchers over to service users (ie, patients) [11]. Stakeholder engagement has been shown to improve the relevance of research, increase stakeholder trust in research and researchers, enhance mutual learning between stakeholders and researchers, and improve research adoption [12,13].

Even with the apparent benefits of PCOR, the PCORI recognizes that before patient–researcher partnerships can work effectively and successfully, some level of initial training for both the researchers and the patient partners is necessary [10]. In 2013, the PCORI stated that patients and stakeholders need training “to have productive conversations with research partners” while researchers need training to “adopt a vernacular that is familiar to patients and stakeholders and facilitates best communication” [10]. We found several studies that evaluated PCOR programs and interactions, findings from which we identified priorities for PCOR training [14-16]. One PCOR training program for patient partners of the National Organization of Rare Disorders (NORD) in conjunction with the University of Maryland included PCOR funding opportunities, use of data sources to help support PCOR partnerships, different levels of patient engagement, and techniques for communication and collaboration [17]. An evaluation of a training program for new PCOR researchers conducted at the University of Maryland suggested the need for qualified and skilled mentors in PCOR methodology [15]. A separate study found that training priorities should include helping team members identify appropriate patient partners, devising an engagement strategy that clarifies roles and expectations, and building skills for positive team dynamics [14]. PCOR training for researchers and patients can support productive relationships that advance patient-centered outcomes research.

To better inform PCOR training, we performed an educational needs assessment to determine the interests and concerns of the CF community. Such assessments are considered fundamental to the success of training programs and to identifying potential gaps and discrepancies between learner types, in this case those between patients/caregivers and researchers/health care providers [18,19]. The aim of this study was to inform the development of a future PCOR training program for the CF community.

## Methods

### Study Design

We developed our needs assessment using a mixed-methods approach, which helps to strengthen a study’s conclusions and provide greater validity to the findings [20]. We designed our needs assessment using both a quantitative survey and qualitative in-depth discussion groups to inquire about the PCOR training needs of the CF community. The survey sought to assess the overall interest of PCOR in the community and to understand respondents’ perceived barriers and concerns about PCOR. We followed up with these findings with 3 in-depth discussion groups, called World Café, to further explore these concepts. In both the survey and discussion sessions, we assessed PCOR knowledge and experience, possible PCOR training program topics, and potential training session formats. This study was provided exempt status by the University of Washington Human Subjects Division (Institutional Review Board no. 6146).

### Patient Involvement

Within the CF community is a well-established organization built upon PCOR principles, the Cystic Fibrosis Reproductive and Sexual Health Collaborative (CFReSHC). Through PCORI pilot funding, CFReSHC was established in 2016 with a team of CF researchers, patients with CF, and reproductive health–trained family physicians, obstetricians, and gynecologists. The aim of this collaborative is to create and maintain an authentic coproduction partnership [21-23]. To accomplish this, the CFReSHC meaningfully engages patients and other stakeholders (such as clinicians, payers, and policy makers) throughout the research process, including the planning, conduct, and dissemination of the research [24-26]. CFReSHC members, including patients with CF, were involved in this and other studies’ design, execution, interpretation of findings, dissemination, and authoring.

### Participants

Our primary objective was to make the survey accessible to as many people as possible in the CF community. We invited a convenience sample of diverse members of the CF community to participate in our needs assessment, including patients with CF, caregivers, all members of the CF clinical care team, researchers, and research staff. Because CF is a complex multi-organ disease, the CF clinical care team is comprehensive and interdisciplinary in nature and consists of clinicians, nurses, respiratory therapists/physical therapists, social workers, and nutritionists. Many CF clinics have additional specialists in gastroenterology, endocrinology, complex pharmacy, advanced practice, occupational therapy, mental health, and care coordination.

### Recruitment

Our initial phase of recruitment involved reaching out to organizations that serve the CF community and spreading the word about the survey through multiple communication platforms and social media channels. We contacted 4 prominent organizations in the CF community, including the CFF, CFRI, CFReSHC, and CF Roundtable. We also used CF community list servers through the CFF and thus reached each of the 130 existing care teams in the United States. Once the responses slowed to less than 1 response per week, we stopped recruitment. We invited survey respondents who provided their contact information to participate in 1 of 3 follow-up 1.5-hour World Café discussion sessions.

### Survey

We developed an anonymous 35-question online survey based on questions from a prior needs assessment conducted through the University of Washington Institute of Translational Health Sciences (ITHS) Community Voices Program, which is a 5-state program that connects community organizations with academic researchers [27]. The questionnaire (Multimedia Appendix 1) included definitions from the PCORI about specific terms, including patient engagement, PCOR, and comparative effectiveness research in order to increase baseline knowledge for the respondents about the questions being asked. Questions included respondent identification to specific self-identified participant groups (patient/caregiver vs researcher/health care provider). We then tailored a few questions to the specified groups based on topic areas deemed as priority areas in other PCOR training sessions [14,15,17]. We asked about participant knowledge of and experience with PCOR, preferred PCOR topics, and formats for additional training and asked participants to rank the importance of their selection using a 4-point Likert scale: “extremely important,” “important,” “not important,” or “not at all important.” We used a 4-point Likert scale to ensure participants selected a nonneutral position and to reduce the complexity of responses, given the breadth of participants from whom we sought input [28,29]. Two questions were mandatory, including the type of participant and interest in participating in a follow-up discussion session, while the other questions did not require responses to move forward. Survey features included a “back” button allowing participants to review and change responses. Participants also had the option to save and return to the survey later using their specific survey link. Our educational specialist and 4 CF community team members reviewed and modified the survey to ensure relatedness and understandability of questions in our target population. Surveys were administered for 7 weeks in November and December of 2018. We collected data through Research Electronic Data Capture (REDCap; Vanderbilt University), an encrypted and secure, HIPAA (Health Insurance Portability and Accountability Act)-compliant, survey database hosted by the University of Washington ITHS [30]. The survey was in English, took approximately 15 minutes to complete, and was voluntary. At the end of the first survey, participants had the option to enter personal information in a second survey if they were interested in participating in a World Café discussion session. The second survey with personal information was not linked to the responses of the first survey.

### Follow-up World Café Sessions

In January 2019, we conducted 3 separate in-depth discussion sessions to further explore survey respondents’ preferences for PCOR training in the CF community. We structured our discussion sessions using the World Café methodology [31]. World Café is a consensus-building community participatory tool designed to allow several small-group conversations to take place at separate tables, with participants systematically rotating to different tables approximately every 20 minutes. World Café provides a setting in which community participants discuss diverse perspectives in order to generate new collective intelligence [32]. World Café was selected as the method with the understanding that the ideas gathered remain in the domain of the participants, not with the researchers. Because of the need for strict infection control in CF and the inability of affected individuals to convene in a single room [33], we conducted our World Café sessions online via videoconferencing using Zoom (Zoom Video Communications, Inc). The online video conferencing software allowed geographically dispersed discussant participants to convene as a single large group and in concurrent, separate, smaller group discussions.

In keeping with World Café guidelines, we started each discussion session with a large-group 20-minute introduction to set the context and create a hospitable space. This included providing a project overview and reviewing the definition of engagement and benefits of PCOR. We then broke the larger group into 2 small-group discussions facilitated by a patient partner–researcher/clinician dyad experienced in PCOR methodology. One team member facilitated the discussion with questions, while the other took detailed notes. Patients/caregiver discussant participants met separately from researchers/health care providers to encourage sincere feedback and to minimize any power dynamics [34]. Each breakout room discussed their questions for 15 to 20 minutes, with facilitators encouraging everyone’s contribution before switching to the other breakout room. We based topic discussion questions on the survey findings from the 170 participants from the CF community. Both breakout rooms discussed participants’ interests in PCOR and motivations to encourage others to participate in PCOR. In one breakout room, participants discussed their concerns about participating in PCOR and likelihood of their attending trainings with other patients or researchers. In the other breakout room, participants discussed important topics and skills to incorporate into PCOR training for the CF community. At the start of each small breakout room discussion, participants were shown the responses to the questions the prior small group discussed, so that the new group was quickly brought up to speed on the issues raised in the prior discussion. At the end of each discussion session, the facilitators simultaneously reviewed the detailed breakout sessions notes and compiled and repeated to the group the ideas that emerged for the participants to review to ensure all ideas discussed during their session were captured. After each breakout room session was complete, both the patient/caregiver and researchers/health care provider groups were convened into a large group and asked to vote for their top 3 responses to each theme to create community consensus and define priorities. Voting was chosen because it allowed the final decision-making to remain in the hands of the participants and created a venue for consensus building after a series of discussions [35]. Incentives were offered to participants who participated in a World Café session.

### Analysis

Because nonresearch stakeholders have not been traditionally part of CF research teams, we assumed the PCOR learning needs of patients and caregivers would be similar, and thus combined our findings for these 2 stakeholder groups. Similarly, we combined the findings of the researchers and health care provider groups as well.

#### Survey

We analyzed complete survey data using descriptive statistics to compare and contrast frequencies of responses between participant groups.

#### Follow-up World Café Sessions

We took the top 4 ideas within each theme as indicated by simple majority vote as priorities for a future PCOR training program. Quotations from the World Café discussion notes provided examples to illustrate and clarify priority areas.

## Results

At the time of survey closure, we had a total of 170 responses. Among the respondents were 96 CF patients and caregivers, and 74 researchers, research staff, and health care providers, all of whom completed the online survey, with 22 participating in the follow-up World Café discussion sessions. We included all participants in the analysis.

### Survey

More than half (52/96, 54%) of patients/caregivers and 86% (64/74) of researchers/health care providers had heard of PCOR, but only about one-third of both groups had ever previously participated in PCOR (Table 1).

**Table 1:** Participant roles and survey responses (N=170).

Participant role and responses			Value, n (%)
**Survey participant role**			
	Patients/caregivers		96 (56)
	**Providers/researchers (n=74)**		74 (44)
		Physician/advanced practice provider	19 (26)
		Nurse	15 (20)
		Other^a^	40 (54)
**Participated in 3 discussion sessions (n=22)**			
	Patients/caregivers		12 (55)
	Providers/researchers		10 (46)
**Previous experiences with PCOR^b^**			
	**Participants who had heard of PCOR**		116 (68)
		Patients/caregivers	52 (54)
		Providers/researchers	64 (86)
	**Participants who had participated in PCOR**		57 (34)
		Patients/caregivers	34 (35)
		Providers/researchers	23 (31)

^a^Other provider/researcher roles included dietitians, respiratory therapists, psychologists, mental health coordinators, social workers, researchers, and research coordinators.

^b^PCOR: patient-centered outcomes research.

Almost 86.5% (147/170) of all respondents were interested in participating in PCOR. Before participating in PCOR, patients/caregivers wanted to understand more about expectations of them as partners on PCOR research teams (82/96, 85%; Table 2). Researchers/health care providers, in contrast, wanted to hear more about how to include outcomes important to patients/caregivers (55/74, 74%) and the quality and impact of PCOR research (52/74, 70% and 51/74, 69%, respectively; Table 2).

**Table 2:** Participant ratings of what to learn before engaging in patient-centered outcomes research.

Participant responses by role		Rating (N=170), n (%)			
		Extremely important	Important	Not important	Not at all important
**Patients/caregivers^a^**					
	What the expectations are^b^	82 (85)	11 (11)	0 (0)	0 (0)
	The benefits to you or your child	60 (62)	28 (29)	3 (3)	3 (3)
	How to share your expertise	59 (61)	29 (30)	6 (6)	0 (0)
	How to partner with researchers	52 (54)	40 (42)	2 (2)	0 (0)
	How to establish open communication	61 (64)	30 (31)	2 (2)	1 (1)
**Researchers/providers**					
	How to include PCOR^c^ outcomes	55 (74)	18 (24)	1 (1)	0 (0)
	The quality of PCOR	52 (70)	19 (26)	2 (3)	0 (0)
	The impact of PCOR	51 (69)	21 (28)	1 (1)	0 (0)
	How to navigate IRB^d^	33 (45)	39 (53)	2 (3)	0 (0)
	How to identify PCOR research topics	48 (65)	23 (31)	3 (4)	0 (0)
	How to design a PCOR study	32 (43)	34 (46)	7 (9)	0 (0)

^a^Two missing values are from respondents misclassified as researchers/providers who did not receive these questions.

^b^One missing value is from a participant who did not respond.

^c^PCOR: patient-centered outcomes research.

^d^IRB: institutional review board.

**Figure 1:**
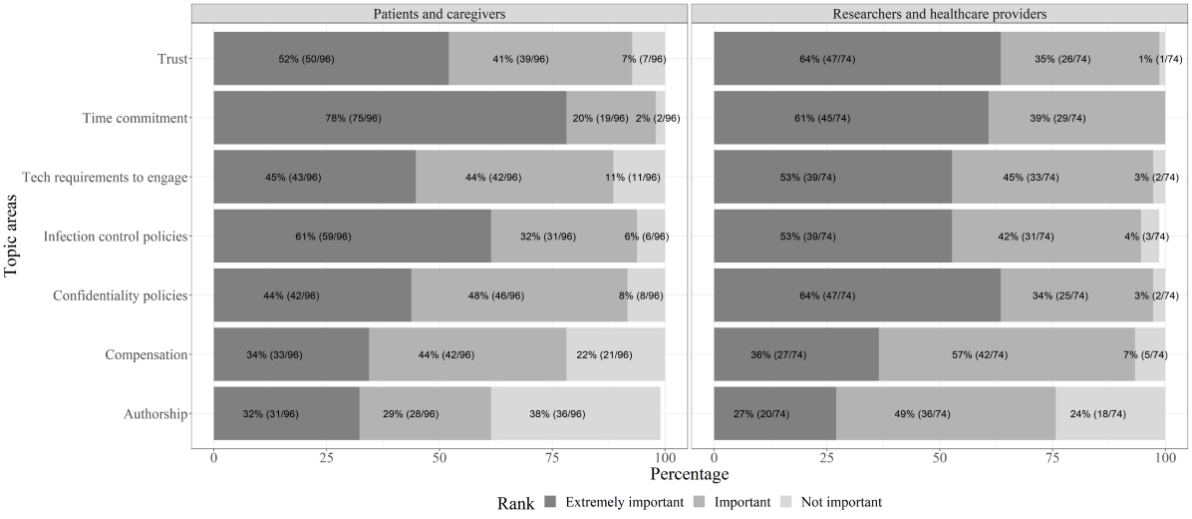
Desired topics to address in patient-centered outcomes research (PCOR) training by participant type.

Desired PCOR training topics and training format differed between the patient/caregiver and researcher/health care provider groups (Figure 1). Patients/caregivers were most interested in learning about the time commitment as a PCOR team member (75/96, 78%). Researchers/health care providers wanted to equally receive training about how to establish trust (47/74, 64%) and maintain confidentiality (47/74, 64%) when patient or caregiver partners are on the PCOR team. The majority of patients/caregivers wanted to learn about PCOR using online interactive sessions (80/96, 83%), whereas researchers/health care providers preferred to have training at their CF center (54/74, 73%) or as a webinar (51/74, 69%; Table 3).

**Table 3:** Session format preferences^a^.

Format	Patients/caregivers (n=96), n (%)	Researchers/providers (n=74), n (%)	Total (N=170), n (%)
Webinar	69 (72.0)	51 (68.9)	120 (70.6)
Onsite training at your CF^b^ center	44 (45.8)	54 (73.0)	98 (57.6)
Online training	80 (83.3)	46 (62.2)	126 (74.1)
Self-directed learning	73 (76.0)	43 (58.1)	116 (68.2)

^a^Formats that participants indicated they were “likely” to participate in.

^b^CF: cystic fibrosis.

### Follow-up World Café Sessions

We categorized the top 4 concerns about participating in PCOR by patients/caregivers and researchers/health care providers into 2 separate categories (Table 4).

#### Concerns About Participating in PCOR

Participants were concerned about ensuring PCOR groups have a diverse representation of patient partners and that everyone on the PCOR team would have a clear idea of their role. Other priorities to address included overcoming the existing power dynamic that exists between patients/caregivers and researchers/health care providers, defining roles, and keeping patient partners engaged throughout the research process.

#### Topics and Skills to Include in a PCOR Training Program

The top 4 topics and skills to include in a PCOR training program for the CF community included the illustration of good PCOR team dynamics (Table 4). For example, participants discussed solutions to this recommendation to include tips such as icebreaker games and team building exercises to help level the hierarchy and allow team members to get to know each other. Other topics participants indicated for a PCOR program included teaching members of the research team how to genuinely appreciate contributions of patient partners and other stakeholders as well as how to construct a transparent process and create a shared language. Participants thought that providing these tips in a reference manual would be important.

**Table 4:** Top 4 priorities related to concerns about engaging in PCOR and PCOR training topics and skills from World Café discussion sessions.

Issue		Quotation to illustrate issue (source)
**Top concerns about engaging in PCOR^a^**		
	How to engage a representative group of patients	“The families who are able to partner with us may represent a more resourced family. Participants that need the most support may be unable to participate.” (researcher/healthcare provider, discussion session 3)
	How to create defined roles for patients/caregivers	“[It’s important for patients to have] a defined role. Sometimes it is not clear to patients why [they] are there and what [they’re] supposed to be doing.” (patient/caregiver, discussion session 1)
	Level the team hierarchy	“Clinicians often can’t really figure out where the patient fits in. Patients often defer to the clinicians because they don’t have the confidence to speak up.” (researcher/health care provider, discussion session 1)
	How to retain patients as team members throughout the research project	“Making sure [patients’] role is meaningful, integral and acknowledged. Make sure it isn’t tokenism. Inviting patients into initial study planning but not including them in later study design or data analysis or acknowledging them in research products [is tokenism].” (patient/caregiver, discussion session 2)
**Top priorities for a PCOR training program**		
	Provide examples to explain PCOR	“[A PCOR 101 training program should include] a combination of personal testimony, to get investigators engaged, and a nuts and bolts manual to refer to after the training is over.” (researcher/provider, discussion session 1)
	Teach how to appreciate all PCOR team members	“Help providers understand that patients are the experts in their own story.” (patient/caregiver, discussion session 3)
	Demonstrate how to construct transparency	“[There is] suspicion in the community because there is no feedback loop and no sustained benefit from coming to the table.” (patient/caregiver, discussion session 3)
	Create a shared language	“[Training] should be done jointly. [I worry] about medical jargon used by medical personnel, but patients know much of that too. [A joint training session] will open up a dialog—doctors would actually hear patient concerns.” (patient/caregiver, discussion session 2)

^a^PCOR: patient-centered outcomes research.

#### Training Logistics

Across all 3 discussion sessions, most participants (4/5, 80%; 3/5, 60%; 7/9, 77%) thought the 2 separate learner groups (patients/caregivers and researchers/providers) should be trained together to mimic PCOR, with patients and researchers/health care providers working as equal members of the research team. They also thought that these 2 learner groups may have specific learning needs for which training could take place separately, such as patients/caregivers needing to learn basic research concepts and researchers/providers needing to learn skills on group facilitation and creating an inclusive working environment. Only 5% (1/19) thought each learner group should be trained entirely as separate groups.

## Discussion

### Principal Findings

We performed this educational needs assessment as a first step in designing a PCOR training program for the CF community. The majority of respondents reported being interested in participating in PCOR. Most participants desired joint patient/caregiver and research/provider learning sessions, except in cases where each group had unique learning needs such as training patients in research fundamentals. Respondents acknowledged that there is currently no formal PCOR training and that such a training program would fill a methodological need for the CF community.

Training priorities expressed by our participants were similar to prior research [14,17]. In our study, both patient/caregiver and researchers/providers wanted to know how to partner with one another, indicating the importance of creating an engagement strategy. In the qualitative study of diverse PCOR team members, Lavallee et al [14] notes that depending on the level at which patient and other stakeholders participate on the team (ie, collaborator vs consultant), researcher training should include tips about taking additional time to build trust, clarifying roles, and ensuring that patient input is not limited once the patient agrees to participate. PCOR training for patient partners conducted by NORD similarly devoted 2 sessions to outlining patient engagement throughout the research project and emphasizing the importance of defining roles by using case examples from rare diseases, including CF [17]. These training sessions included team dynamics such as transparency, bidirectional learning, and developing a structure for collaboration [14,17], which were also highlighted by our World Café participants. Our follow-up World Café discussions emphasized the importance of providing training that shows “how” to partner with one another, similar to how the University of Maryland has used experienced PCOR mentors for new PCOR teams [15]. PCOR guidelines developed by the American Thoracic Society (ATS), a professional organization to which many CF specialists belong, also touch on supporting new PCOR teams with “how” to do PCOR by recommending that researchers create a mechanism in which to share lessons with one another [36].

Building upon prior research, our finding highlighted concerns about apparent hierarchical issues that exist between clinicians/researchers and patients in the CF community. The ATS guidelines suggest the importance of teams developing processes in which perspectives are balanced to help reach consensus and fostering a collaborative spirit from the start, thus mitigating hierarchy [36]. Through our literature review, we found that PCOR teams that were deemed to be successful included members with excellent facilitation and communication skills and incorporated evidence-based strategies to achieve the teams’ aims and outcomes [11]. Similarly, a mixed methods study of PCORI pilot project awardees found that successful PCOR teams require strong relationships between members, engagement expertise, and institutional leadership that supports PCOR [37]. A separate qualitative study consisting of a large hospital research collaborative also stressed the importance of building supportive environments between patients, families, and researchers [38]. Despite not being specifically mentioned by our participants, a number of articles underscore the importance of including financial support for researchers specifically to build and maintain PCOR teams [37,39].

The CF community is well positioned to build capacity in PCOR, and even prior to the era of COVID-19, people with CF were well versed in engaging online. This study successfully demonstrates the ability to incorporate community engagement and mixed methods research virtually. Additionally, previously mentioned, many people with CF and their caregivers are already active in stakeholder-sponsored research events through patient advocacy organizations such as the CFF and CFRI. The CFF currently trains and onboards new patient partners and family members in clinical quality improvement work. In organizations like the CFReSHC, patients with CF are self-stewards of research proposal ideas, and they partner with academicians, stakeholder organizations, and health care providers as equals on research teams. The members of the CFReSHC are both willing and well situated to assist research teams new to PCOR with coaching and guidance as needed.

### Limitations

There were several limitations to this study. Due to our community-wide recruitment, we are unable to report our actual response rate. Rather, our findings are limited to a convenience sample and may not be representative of the learning needs or desires of everyone in the CF community. Additionally, our World Café discussion group participants were limited to those who responded to the survey. We do not believe this was a critical flaw because the survey opportunity likely helped build rapport with the researchers and allowed us to select participants who were eager to reflect on various issues in PCOR [40]. Follow-up questions to further assess learning gaps are needed to further confirm our findings.

### Conclusions

The majority of respondents in the CF community are interested in PCOR. A PCOR training program would fill a current methodological research gap in the CF community. The results of this needs assessment were used to create a pilot PCOR training program for the CF community.

## Appendix

Multimedia Appendix 1 Online needs assessment survey for the cystic fibrosis community.

## Abbreviations

ATS: American Thoracic Society
CF: cystic fibrosis
CFF: Cystic Fibrosis Foundation
CFReSHC: Cystic Fibrosis Reproductive and Sexual Health Collaborative
CFRI: Cystic Fibrosis Research, Incorporated
HIPAA: Health Insurance Portability and Accountability Act
ITHS: Institute of Translational Health Sciences
NORD: National Organization of Rare Disorders
PCOR: patient-centered outcomes research
PCORI: Patient-Centered Outcomes Research Institute
REDCap: Research Electronic Data Capture
