# Supplementary material for: Educational Needs and Preferences for Patient-Centered Outcomes Research in the Cystic Fibrosis Community: Mixed Methods Study
Source: JMIR Form Res. 2021 Mar 4;5(3):e24302. doi: 10.2196/24302 (PMC7974760; doi:10.2196/24302)
Supplement: Multimedia Appendix 1 [file formative_v5i3e24302_app1.pdf]

PCOR Needs Assessment Survey

The following is a Patient-centered Outcomes Research needs assessment survey for Cystic Fibrosis researchers, clinicians, patients and caregivers. Our goal is to better understand your knowledge and experience with patient-engaged research (or PCOR) and determine training topics most important to you. This survey is being conducted by the Cystic Fibrosis Reproductive and Sexual Health Collaborative (CFReSHC) which is an innovative collaborative that engages CF patients as key members of the research team. CFReSHC received funding from the Patient-Centered Outcomes Research Institute (PCORI) to expand our patient-engaged methods to the greater CF community. Your input about PCOR training is very important to us.

Your participation in this survey is voluntary. You may refuse to participate and you are free to stop taking the survey at any time without penalty or loss of benefits to which you are otherwise entitled.

If you have questions about the survey, please contact Dr. Emily Godfrey at (206) 685-4895. If you have questions about your rights as a research subject, please contact the University of Washington Human Subjects Division at (206) 543-0098.

As you respond to the questions below, please keep the following statements in mind:

Patient engagement: meaningful involvement of patients, caregivers, clinicians, and other healthcare stakeholders as equal members throughout the research process - from topic selection through design and conduct of research to dissemination of results.

Patient-centered outcomes research: studies questions and outcomes that are meaningful and important to patients and caregivers.

Patient-engaged research: patients are equal partners with researchers and health care providers in the research process.

Comparative clinical effectiveness research (CER): compares the effectiveness of two or more interventions or approaches to health care, examining their risks and benefits. CER findings can both validate a particular intervention and identify which treatments best meet a certain population's needs.

Please select the answer that best describes you:<br>*must provide value	◯ Person with CF or Caregiver of a person with CF<br>◯ CF care provider, team member, or researcher
Have you heard about patient-engaged research, before this survey? ^	◯ Yes<br>◯ No
Have you ever participated in patient-engaged or patient-centered outcomes research? ^	◯ Yes<br>◯ No
Please select the best description of your current position: +<br>*must provide value	◯ Physician<br>◯ Advanced Practice Provider<br>◯ Nurse<br>◯ Dietitian<br>◯ Respiratory Therapist<br>◯ Psychologist<br>◯ Social Worker<br>◯ Researcher<br>◯ Research Coordinator<br>◯ Administrator<br>◯ Other
Please specify other description: + [Question only shown with a selection of "other" in the previous questions]<br>*must provide value	__________________________________
Have you ever participated in research? +	◯ Yes, as an investigator<br>◯ Yes, to help recruit patients<br>◯ No<br>◯ Other

Before today, have you heard about patient-engaged or patient-centered research? +	◯ Yes<br>◯ No
Have you ever participated in patient-engaged or patient-centered outcomes research? +	◯ Yes<br>◯ No

How important is learning about each of the following patient-engaged methods to you?

Patient engagement: meaningful involvement of patients, caregivers, clinicians, and other healthcare stakeholders as equal members throughout the research process.

Patient-centered outcomes research: studies questions and outcomes that are meaningful and important to patients and caregivers.

Patient-engaged research: patients are equal partners with researchers and health care providers in the research process.

	Extremely Important	Important	Not Important	Not at All Important
How to partner with researchers ^	○	○	○	○
How to share your expertise on a research team throughout the research process ^	○	○	○	○
How to build trust with researchers and clinicians ^	○	○	○	○
How to have open communication on a research team ^	○	○	○	○
How to participate in grant writing ^	○	○	○	○
How to participate in publishing and sharing study results ^	○	○	○	○
How to partner with patients, caregivers or CF advocates +	○	○	○	○
How to design a patient-engaged research study +	○	○	○	○
How to identify and select research topics that matter to patients +	○	○	○	○
How to include outcomes that patients or caregivers care about (such as survival, function, symptoms, and quality of life) +	○	○	○	○
How to learn about agencies that fund patient-engaged research +	○	○	○	○
How to develop a comparative effectiveness research question +	○	○	○	○
How to develop a comparative effectiveness research protocol +	○	○	○	○

How important are the following to you before participating in patient-engaged research?

Patient-engaged research: patients are equal partners with researchers and health care providers in the research process.

	Extremely Important	Important	Not Important	Not at All Important
Knowing the time commitment when patients/caregivers are on the research team +	○	○	○	○
Knowing the technology requirements needed for online engagement when patients/caregivers are on the research team +	○	○	○	○
Knowing how to navigate IRB when patients/caregivers are on the research team +	○	○	○	○
Knowing how to maintain confidentiality when patients/caregivers are on the research team +	○	○	○	○
Knowing how to develop and maintain trust when patients/caregivers are on the research team +	○	○	○	○
Knowing the importance of compensation or other benefits when patients/caregivers are on the research team +	○	○	○	○
Knowing about authorship or other career development opportunities related to research when patients/caregivers are on the research team +	○	○	○	○
Knowing the infection control policies when patients/caregivers are on the research team +	○	○	○	○
Knowing the QUALITY of research when patients/caregivers are on the research team +	○	○	○	○

Knowing the IMPACT of the research when patient/caregivers are on the research team +	◯	◯	◯	◯
Knowing the time commitment ^	◯	◯	◯	◯
Knowing what is required of me ^	◯	◯	◯	◯
Knowing the technology requirements needed for online engagement ^	◯	◯	◯	◯
Knowing the confidentiality practices and/or policies ^	◯	◯	◯	◯
Knowing the compensation or other benefits ^	◯	◯	◯	◯
Knowing the infection control policies ^	◯	◯	◯	◯
Knowing how to develop and maintain trust with fellow patients/caregivers and researchers/providers ^	◯	◯	◯	◯
Knowing about authorship or other career development opportunities ^	◯	◯	◯	◯
Knowing the benefits to you or your child ^	◯	◯	◯	◯

Do you have any comments or additional topics you would want to know about before participating in patient-engaged research?

____

Would you be interested in participating in patient-engaged research?

◯ Yes
◯ No

Why?

____

How likely are you to participate when the trainings are delivered in the following ways?

	Likely	Neutral	Unlikely
Webinars	○	○	○
Onsite training in your CF center	○	○	○
Online training	○	○	○
Self-directed learning (learning on your own at your own time using instructional videos or podcasts)	○	○	○

For the patient-engaged research training specifically, how likely would you be to attend a training (together) with the following individuals?

Patient-engaged research: patients are equal partners with researchers and health care providers in the research process.

	Likely	Neutral	Unlikely
CF Researchers	◯	◯	◯
CF Healthcare Providers	◯	◯	◯
CF Patient Partners	◯	◯	◯
CF Caregiver Partners	◯	◯	◯
CF Advocacy Organization (e.g., CFF or CFRI) Employees	◯	◯	◯

Do you have any other thoughts or comments?

____

Are you interested in participating in a 2-hour online discussion session to share your thoughts about how to create a training program about patient-engaged research for CF clinicians/researchers and CF patient/caregivers? +
*must provide value

◯ Yes
◯ No
(Participants will receive a $100 Amazon gift card for their time.)

Are you interested in participating in a 2-hour online discussion session to share your thoughts about how to create a training program about patient-engaged research for CF clinicians/researchers and CF patient/caregivers? ^
*must provide value

◯ Yes
◯ No
(Participants will receive a $50 Amazon gift card for their time.)

Please provide your email address for us to contact you about the discussion session: [Question not shown with a "no" in either of the two previous questions]
*must provide value

____

This program is funded through a Patient-Centered Outcomes Research Institute (PCORI) Eugene-Washington PCORI Engagement Award (10569-UWash).

Thank you for completing the needs assessment survey!!

^ Question only shown if participant self-selected as "person with CF or caregiver of a person with CF"
+ Question only shown if participant self-selected as "CF care provider, team member, or researcher"
